# Intratumoral *Lactobacillus iners* as a poor prognostic biomarker and potential therapeutic target for cervical cancer

**DOI:** 10.3389/fcimb.2024.1469924

**Published:** 2024-12-20

**Authors:** Yang Liu, Lei Cao

**Affiliations:** ^1^ Department of Obstetrics and Gynecology, Wuxi People’s Hospital, The Affiliated Wuxi People’s Hospital of Nanjing Medical University, Wuxi Medical Center, Nanjing Medical University, Wuxi, China; ^2^ Department of Pathology, Xiang’an Hospital of Xiamen University, Xiamen University, Xiamen, China

**Keywords:** cervical cancer, intratumoral bacteria, poor prognosis, biomarker, therapeutic target

## 1 Introduction

Cervical cancer is the fourth most common cancer among women worldwide, and its occurrence linked to a variety of factors, including human papillomavirus (HPV) infection, genetic predisposition, lifestyle factors, and the composition of the host microbiome (Abu-Rustum et al., 2023). Recently, there has been a growing body of research that examines the influence of the microbiome on the microenvironment of cervical cancer. The microbial ecology of cervical cancer is an intricate ecosystem, whereby *Lactobacillus iners* (*L. iners*) have a notable impact. *L. iners* in the vagina synthesizes hydrogen peroxide through the breakdown of carbohydrates. This process not only hinders the growth of harmful microorganisms, but also exhibits possible damaging effects on tumor cells. Hydrogen peroxide can dismantle the cell membranes of tumor cells, provoke oxidative stress within the cells, and finally result in the demise of tumor cells. In addition, hydrogen peroxide can stimulate the host’s immune system, hence increasing immune surveillance against tumor cells (Castanheira et al., 2021).

**Figure 1:**
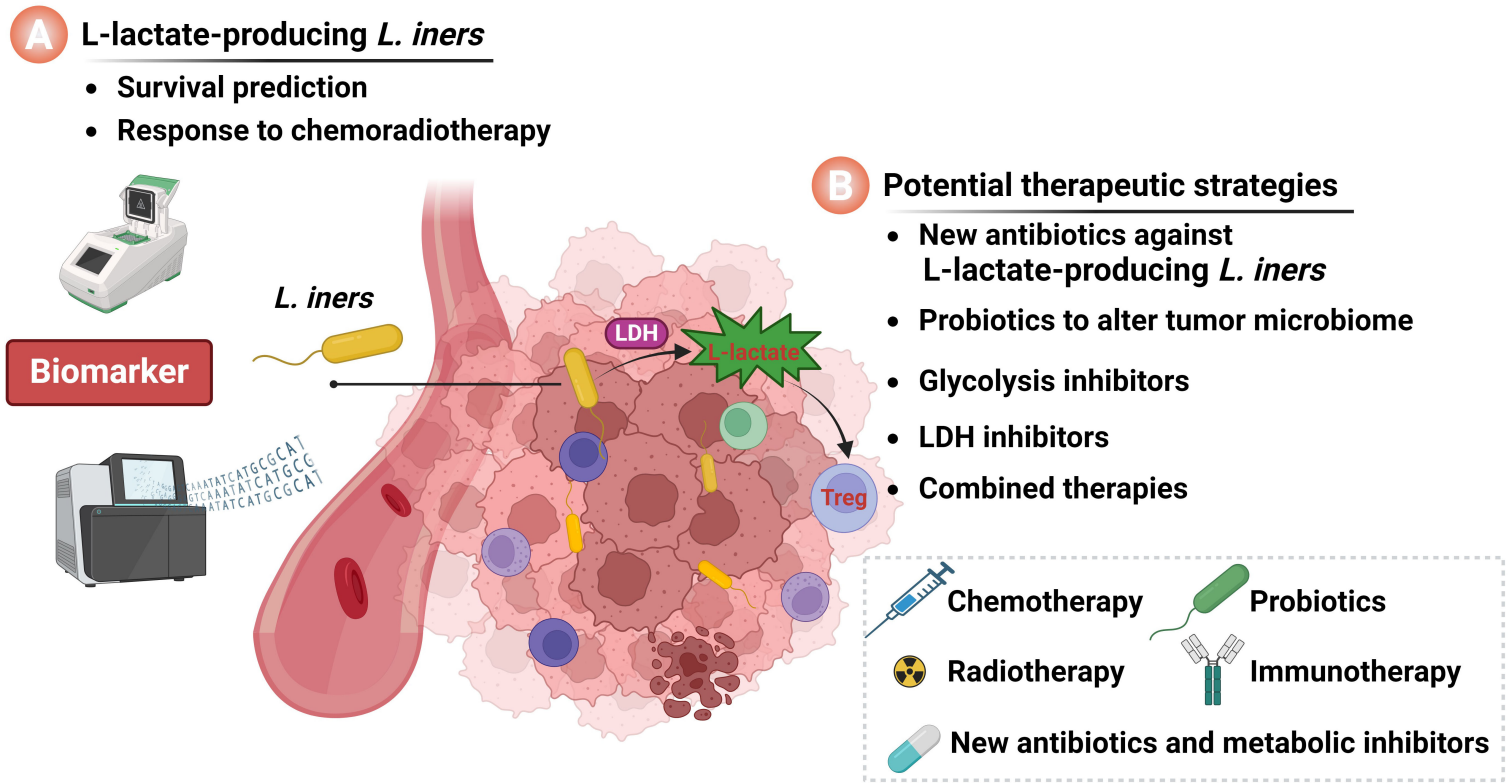
The significance of intratumoral *L. iners* in cervical cancer. **(A)** L-lactate-producing *L. iners* act as a novel prognostic biomarker for cervical cancer patients. **(B)** Intratumoral *L. iners* and their regulatory networks serve as targets for the development of novel therapeutic strategies for cervical cancer. Image was created by BioRender.com.

However, Colbert et al. found that *L. iners* colonizing within cervical cancer tumors can induce metabolic reprogramming of the tumor by producing L-lactate, thereby promoting resistance to radiotherapy and chemotherapy (Colbert et al., 2023). This study provides a new perspective on the biological significance of the tumor microbiota in cervical cancer and warrants further investigation. This opinion article discusses the clinical significance of L-lactate-producing *L. iners* in cervical cancer and proposes that *L. iners* can serve as a novel prognostic marker for cervical cancer (**Figure 1A**). It also presents the viewpoint that by targeting intratumoral *L. iners* and their regulatory signaling networks in cervical cancer, it is possible to design new therapeutic strategies, providing guidance for combination therapies for cervical cancer (**Figure 1B**). As future research progresses, the elucidation of the exact relationship between the tumor microbial environment and tumor progression is expected to offer scientific guidance for the treatment of cervical cancer.

## 2 Intratumoral *L. iners* as a biomarker of poor prognosis in cervical cancer

In the study by Colbert, the analysis of tumor microbiome sequencing in cervical cancer patients treated with radiotherapy and chemotherapy found that patients with *L. iners* residing in their tumors had a poorer recurrence-free survival compared to those without *L. iners* in the tumor, even in cases of smaller tumors such as stage I-II. This suggests that the presence of *L. iners* within cervical cancer tumors has the potential to serve as a biomarker of poor prognosis in patients. Furthermore, this study revealed that the L-lactate-producing *L. iners* can induce resistance in cervical cancer tumor cells to radiotherapy and chemotherapy, including regimens with gemcitabine, cisplatin, and 5-fluorouracil, as well as combined chemoradiotherapy. In-depth mechanistic analysis indicated that the chemoradiotherapy resistance induced by *L. iners* was primarily achieved through the production of substantial amounts of L-lactate. RNA-sequencing results demonstrated that the significantly differentially expressed genes regulated by *L. iners* were predominantly enriched in pathways such as hypoxia-inducible factor 1 (HIF-1) transcriptional targets, FGFR signaling, Her2/ERBB2 signaling, and p53/p73-dependent apoptotic signaling. These pathways are closely associated with lactate signaling and lactate dehydrogenase activity. L-lactate is also an important marker of aberrant tumor glucose metabolism. Colbert et al. examined how *L. iners* influences the metabolic function of cervical cancer tumors. The researchers discovered that tumors positive for *L. iners* showed more pronounced glycolytic traits and significant changes in their metabolic profile, including modifications in the metabolism of glutathione, urea, and different amino acids. These findings emphasize the intricate influence that the microbiome may have on the advancement and response to treatment of cervical cancer, emphasizing the need for additional research in this field to comprehend the underlying mechanisms and investigate potential therapeutic approaches that could enhance patient outcomes (Johnston and Bullman, 2024).

A thorough and comprehensive methodology is required to establish *L. iners* as a clinically significant biomarker for prognosis and therapy stratification in cervical cancer. The method commences with extensive validation studies to verify the initial findings over a wide range of patient populations, guaranteeing that the predictive value of the biomarker is strong and applicable to many scenarios. Ensuring uniformity in detection procedures is of utmost importance, necessitating the refinement of DNA extraction methodologies and molecular techniques such as PCR to attain reliable and precise detection results across different laboratories. Establishing the prognostic importance of *L. iners* requires correlating its presence with clinical outcomes. This requires rigorous statistical analysis to account for any variables that could influence the results and to accurately measure the biomarker’s ability to predict outcomes. Subsequent prospective cohort studies are necessary to monitor patients over a period, offering empirical evidence of the usefulness of *L. iners* in forecasting the advancement of diseases and the effectiveness of treatments.

## 3 Intratumoral *L. iners* as a potential therapeutic strategy to enhance sensitivity in cervical cancer treatment

The identification of *L. iners* within cervical cancer tumors presents a new and promising focus for cervical cancer treatment. Colbert’s research showed that the presence of *L. iners* in cervical cancer patients is linked to a negative outlook, including an elevated likelihood of recurrence after radiochemotherapy. *L. iners* promote resistance to radiochemotherapy in tumor cells through the abundant production of L-lactate. This process involves many signaling pathways that are strongly linked to tumor metabolism and survival (Colbert et al., 2023). As a result, there are many therapeutic approaches that focus on attacking *L. iners*. One effective approach to overcoming resistance to radiochemotherapy is to produce medications that restrict the growth of *L. iners*, a bacterium that specializes in generating L-lactate. Possible pharmaceutical options may consist of novel antibiotics, inhibitors that specifically target metabolic pathways of *L. iners*, or innovative medicines that manipulate the tumor microbiome environment, such as probiotics that can modify the makeup of tumor microbiota and decrease the prevalence of *L. iners* strains that are resistant. Additionally, utilizing synthetic biology techniques to hinder the production of L-lactate by *L. iners*, which in turn disrupts signaling pathways like glycolysis and alters the metabolic microenvironment of tumors, offers a promising avenue for treating cervical cancer. Considering that *L. iners*, specialized in producing L-lactate, can extensively induce changes in cervical cancer metabolic characteristics, particularly the glycolytic oncogenic signaling pathway, the use of glycolytic inhibitors is also a potential strategy to enhance the efficacy of radiochemotherapy in cervical cancer. Glycolytic inhibitors like 2-deoxy-D-glucose (2-DG) have been extensively studied in various tumor treatments and can reduce the energy supply of tumor cells by inhibiting glycolysis, thereby achieving the goal of “starving tumors.” 2-DG inhibits the activity of glucose-6-phosphate isomerase (GPI) by competitively binding with glucose, thereby reducing the production of L-lactate (Su et al., 2023). Additionally, 3-bromopyruvate (3-BrPA) demonstrates notable antitumor effects by inhibiting hexokinase (HK) activity and blocking the glycolysis pathway (Yang et al., 2019). Nevertheless, glycolysis inhibitors encounter multiple obstacles in their therapeutic utilization. First and foremost, glycolysis is an essential metabolic route for regular cells, and if glycolysis is inhibited throughout the body, it can result in significant adverse effects. Furthermore, tumor cells possess a significant ability to adapt their metabolism and can counteract the suppression of glycolysis by utilizing alternate metabolic pathways, such as oxidative phosphorylation. Hence, while treating cervical cancer with *L. iners*-positive tumors, it is crucial to carefully evaluate issues such as biosafety, bioavailability, and targeted delivery when using a combination of glycolysis inhibitors (Cheng et al., 2022). In addition, it is vital to mention that disrupting the function of *L. iners* to suppress the formation of L-lactate is also a technique to deprive tumors of nutrients, since L-lactate serves as a crucial source of energy for tumor cells.

Developing therapeutic techniques that specifically focus on the metabolic route of L-lactate is also a feasible option. L-lactate is generated by the process of pyruvate reduction, which is facilitated by the enzyme lactate dehydrogenase (LDH). LDH has significant upregulation in cervical cancer cells, where it facilitates the enzymatic conversion of pyruvate into L-lactate. Research has furthermore discovered that there is a strong correlation between elevated levels of LDH and the advancement and growth of cervical cancer (Jia et al., 2024). Thus, LDH is seen as a promising target for anticancer therapies, specifically focusing on disrupting the L-lactate metabolism of cervical cancer by LDH targeting techniques. Scientists are presently working on the development of other LDH inhibitors, including Galloflavin and FX11. These inhibitors decrease the synthesis of L-lactate by obstructing the function of LDH, thus impeding the proliferation and dissemination of cancerous cells (Pradenas et al., 2023). For example, FX11 has been discovered to specifically block LDHA, reduce the formation of L-lactate, and demonstrate substantial anticancer effects in several tumor types (Boufaied et al., 2024). Moreover, prior research has demonstrated that the synergistic utilization of LDH inhibitors in conjunction with other therapeutic strategies can augment the antitumor efficacy. For instance, the concurrent use of LDH inhibitors with radiation therapy can enhance the susceptibility of tumor cells to radiation, thus enhancing the effectiveness of treatment (Han et al., 2023). Furthermore, certain research suggest that L-lactate hinders the body’s immune response to tumors by reducing the functioning of T cells and encouraging the development of regulatory T cells (Hayes et al., 2021). Therefore, the combination of LDH inhibitors and immunotherapy is a highly effective treatment strategy. Immune checkpoint inhibitors augment the effectiveness of T cells in combating tumors by obstructing the pathways that allow the immune system to evade detection. This combination therapy can enhance the immune responses against tumors through many pathways, hence boosting the effectiveness of the treatment. The intricate function of L-lactate-producing *L. iners* in the tumor microenvironment and its potential as a target for therapy has emerged as a prominent subject in cervical cancer research. Through a comprehensive examination of the metabolic pathways of L-lactate and its regulatory mechanisms, it is possible to develop novel inhibitors of glycolysis and LDH. When used in conjunction with other therapy modalities, these inhibitors may provide novel approaches for clinical practice. Although encountering several obstacles, the current research offers a compelling rationale to suggest that directing efforts towards L-lactate-producing *L. iners* could lead to significant advancements in the treatment of cervical cancer.

## 4 Discussion

The occurrence and function of *L. iners* in the microenvironment of cervical cancer have attracted considerable attention. *L. iners* is a crucial element of the vaginal microbiome, as it produces lactic acid to preserve the acidic pH of the vaginal environment. This, in turn, helps prevent the growth of harmful microbes and ensures the overall health of the reproductive tract (Zheng et al., 2021). This acid-producing ability is one of the key reasons for *L. iners*’ importance in human health. In the setting of cervical cancer, *L. iners* and its production of L-lactate may play two roles. On one side, by preserving the acidic environment of the vagina, *L. iners* aid to suppress possible harmful microbes, including high-risk HPV strains linked to an elevated risk of cervical cancer (Shi et al., 2022). Thus, by reducing the likelihood of HPV infection, *L. iners* and its metabolic metabolites may indirectly lessen the risk of cervical cancer. However, new research indicates that individuals with cervical cancer may have a very different vaginal microbiome than healthy women, with less *L. iners* present in both abundance and diversity. The pathophysiology of cervical cancer may be associated with microbial imbalance. There may be a more intricate connection between the control of the tumor microenvironment and the synthesis of L-lactate. L-lactate influences the metabolism of tumor cells in cervical cancer, but it also modulates immunological responses that may impact the tumor development. Furthermore, L-lactate synthesis may change the pH of the tumor microenvironment, which could impact the effectiveness of treatments and the resistance of tumor cells to them. As a result, there is strong evidence to support the complex and crucial role that *L. iners* plays in cervical cancer. Fully understanding its regulatory network within the tumor and developing novel and efficient prognostic tools for cervical cancer based on this understanding are key. These tools can guide clinical treatment of cervical cancer and aid in the development of new therapeutic strategies. This work not only helps us better understand the pathogenesis of cervical cancer but also enables us to provide more precise and effective treatment options for patients, thereby improving treatment outcomes and the quality of life.
